# The Reproducibility of Reference Landmarks in the External Acoustic Meatus (EAM) on Cone Beam Computed Tomography (CBCT) Images

**DOI:** 10.3390/jcm13144226

**Published:** 2024-07-19

**Authors:** Fernanda Sanders-Mello, Ronald E. G. Jonkman, Ynke Baltussen, Frederik R. Rozema, Jan Harm Koolstra

**Affiliations:** 1 Department of Orofacial Anatomy, Academic Centre for Dentistry Amsterdam (ACTA), University of Amsterdam and VU University, 1081 Amsterdam, The Netherlandsj.koolstra@acta.nl (J.H.K.); 2 Department of Orthodontics, Academic Center for Dentistry Amsterdam (ACTA), 1081 Amsterdam, The Netherlands; 3 Department of Oral Medicine, Academic Centre for Dentistry Amsterdam (ACTA), University of Amsterdam and VU University, 1081 Amsterdam, The Netherlands; fred.rozema@acta.nl; 4 Department of Oral and Maxillofacial Surgery, Amsterdam UMC, University of Amsterdam, 1012 Amsterdam, The Netherlands

**Keywords:** diagnostic imaging, cone beam computed tomography, three-dimensional imaging, porion, spatial orientation, external acoustic meatus, anatomic reference

## Abstract

**Objective:** The aim of the present study is to identify a more reliable reference point in three-dimensional cephalometric analysis to replace the Porion point used in two-dimensional analysis, enhancing the accuracy of assessments. **Methods:** The methodology assessed potential alternative landmarks for three-dimensional cephalometric analysis. Utilizing a segmenting technique, anatomical landmarks were accurately pinpointed from the external acoustic meatus of 26 Cone Beam Computed Tomography (CBCT) scans. These landmarks were chosen for their clear and unambiguous detectability. To assess reproducibility, each landmark was replicated twice with a one-week interval by a master’s student. Reproducibility was quantitatively evaluated by analyzing the absolute difference per axis. **Results:** Five possible candidate landmarks were identified: the most anterior, posterior, superior, and inferior points of the external acoustic meatus (EAM) and a notch delineating the epitympanic recess. The reproducibility of pinpointing these landmarks ranged from 0.56 mm to 2.2 mm. The absolute mean differences between measurements were 0.46 mm (SD 0.75) for the most anterior point, 0.36 mm (SD 0.44) for the most posterior point, 0.25 mm (SD 0.26) for the most superior point, 1.11 mm (SD 1.03) for the most inferior point, and 0.78 mm (SD 0.57) for the epitympanic notch. **Conclusions:** The most superior point of the EAM might successfully replace the Porion as an anatomical reference.

## 1. Introduction

Computed Tomography (CT) in dentistry is limited by its high radiation dose, high cost, and mechanical complexity [1]. However, the introduction of Cone Beam Computed Tomography (CBCT) for dento-maxillofacial imaging has enabled scans that are faster and cheaper and have lower radiation levels than regular CT scanners [2,3]. CBCT scans are now widely used in dental fields such as Orthodontics and Oral and Maxillofacial Surgery. As technology advances and radiation doses are reduced, CBCT scans will become more popular due to their improved safety and enhanced diagnostic capabilities.

**Figure 1:**
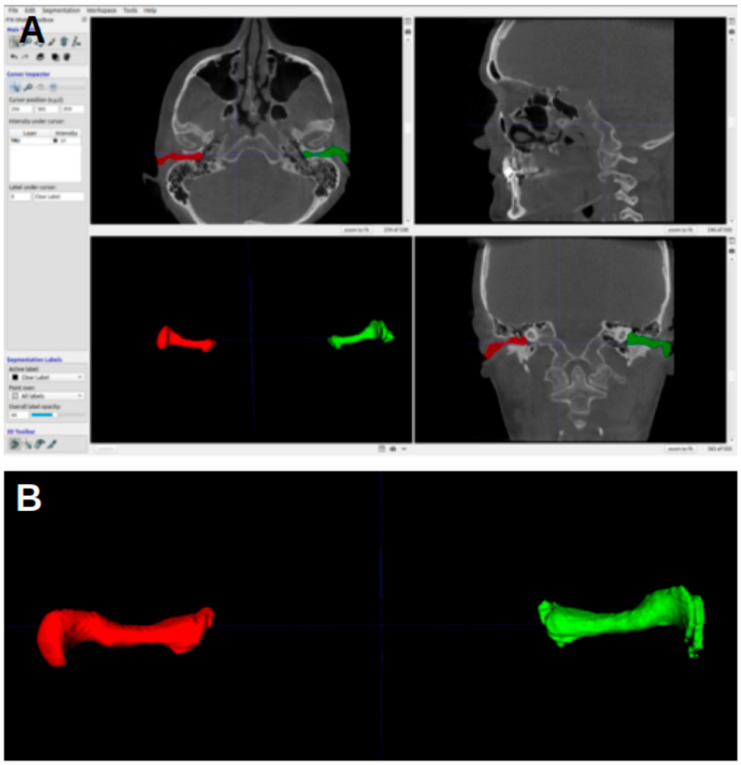
Three-dimensional reconstruction of the acoustic meatus. (**A**) A representation in ITK-snap. (**B**) Coronal view of the segmentations of the right (red) and left (green) meatus, with the EAM flowing into the cauliflower-like shape representing the internal acoustic meatus.

In orthodontics, radiographs are used to plan treatment. They usually involve cephalometric analysis, in which measurements are made based on different cephalometric landmarks [4]. The transition from two-dimensional (2D) to three-dimensional (3D) imaging has created some difficulties for cephalometric analysis, as not all landmarks that have been defined in 2D imaging can easily be converted to 3D [5]. One such landmark is the Porion, which is very often used to determine the Frankfort Horizontal plane [6]. In 2D imaging, the Porion is based on an over-projection of the two acoustic meatus. The cephalometric landmark is an estimation of the Porion’s true location in the external acoustic meatus (EAM), as there is no definitive anatomical point for the Porion in three dimensions. In 3D imaging, determining the Porion point is challenging because the external acoustic meatus (EAM) is a highly irregular structure. Unlike in 2D cephalometric analysis where the Porion is a radiographic point dependent on the projection of X-rays, it is not an anatomical landmark that can be consistently identified in three dimensions. The EAM is approximately three to four centimeters long, varies in shape (Figure 1), and is usually described as an S-shaped form [7]. This shape means that the location of the Porion is not very clear in CBCT images. In this case, the 2D definition does not translate to a landmark in the 3D images.

While most studies define the Porion as the uppermost point of the (right or left) external acoustic meatus [8,9,10], others try to reproduce its 2D location by placing the landmark in the outermost posterior surface of the external acoustic meatus, where the curvature starts [11]. In 3D imaging, this point lies on a surface that is curved in the anterior–posterior direction. As this surface is also curved in the lateral–medial direction, where it penetrates the temporal bone, the Porion is one of the more difficult anatomical landmarks to locate unambiguously on CBCT scans. It may also vary from right to left [12,13].

A systematic review of the reliability and reproducibility of cephalometric landmarks on CBCT scans also found that the Porion landmark had a low level of reliability. Therefore, the aim was to identify a reproducible and accurate anatomical landmark in the acoustic meatus in CBCT imaging that could be used in 3D cephalometry as an alternative to the Porion.

## 2. Material and Methods

This study used 26 CBCT scans previously made for a case–control study of hypermobility disorders [14]. The research protocol for that initial study, which was designed according to the Helsinki declaration with protocol number NL18726.029.07, had been approved by the Medical Ethical Committee at the Vrije Universiteit Medical Center (VUmc), Amsterdam.

The study included subjects aged 18 to 61 years, 8 men and 18 women, with no severe general health impairment, complicated dental abnormalities, or osteoarthritis of the jaw joint [14].

The following scan settings had been used on a NewTom 5G CBCT scanner (QR Verona, Verona, Italy): 110 kVp; 38.25 (range 22.35–55.76) mAs; exposure time of 3.6 s; scan field of view 18 × 12 cm. The isotropic voxel size was 0.3 mm. The subjects were placed in a supine position with their head oriented in the vertical plane with the occlusal plane pointing upwards.

The CBCT images were represented on the computer as DICOM (Digital Imaging and Communication in Medicine) files. The CBCT images were analyzed using ITK-SNAP software, version 3.8, University of Pennsylvania, Philadelphia, PA, USA, (www.itksnap.org, (accessed on 17 June 2023)) [15]. The acoustic meatus were segmented using the “active contour segmentation mode”, which enabled ITK-SNAP to differentiate between different structures according to the grayscale of the image. The segmented acoustic meatus on the right side were labeled with a red color (label 1), and those on the left with a green color (label 2). Figure 1 shows an example of the segmentations.

**Figure 2:**
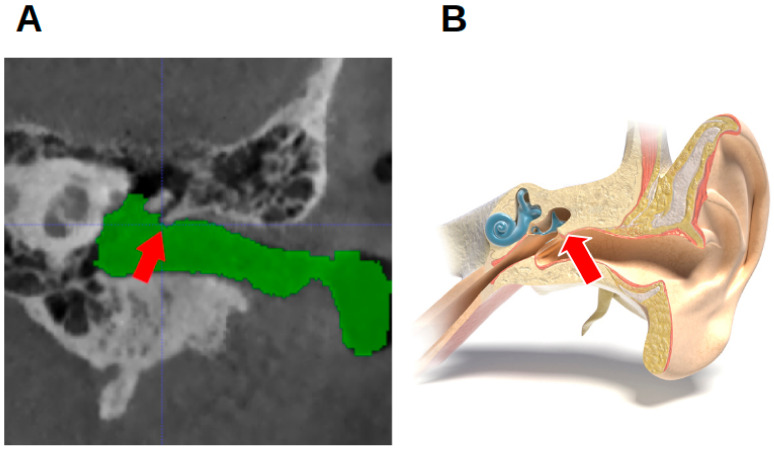
The location of the epitympanic notch. (**A**) A CBCT image of a right acoustic meatus in a coronal view. The red arrow indicates the epitympanic notch. (**B**) A cross-section through the external acoustic meatus (“MedicalGraphics–Drawing Ear anatomy–no labels” at AnatomyTOOL.org by www.MedicalGraphics.de; license: Creative Commons Attribution-NoDerivatives).

Per individual, five landmarks were established in the left and right external acoustic meatus: the most anterior point (MAP); the most posterior point (MPP); the most superior point (MSP); the most inferior point (MIP); and the notch-like rim between the innermost portion of the EAM and the epitympanic recess. As this has no official name, it is referred to as the epitympanic notch (EN) (Figure 2). Angular structures were sought because they might be easier to reproduce [16].

Taking due account of the size of the CBCT voxels (0.3 mm), the coordinates of possible candidates for landmarks were defined according to the Neuroimaging Informatics Technology Initiative (NIfTI). The measurements referred to above were performed twice by a single examiner, a dental student. The second measurement was performed at least a week after the first. The coordinates were used to calculate the absolute difference between the first and second measurement per axis. The accuracy of the measurements was determined based on the difference in millimeters between the first and second coordinate measurements.

Using the same system, the mean anteroposterior (AP), caudal–cranial (CC), and mediolateral (ML) coordinates were then calculated from the location of the same landmark on the same subject. The absolute differences in the anatomical landmarks were used to calculate the mean and the standard deviations per axis. These numbers were used to indicate the reliability of the relevant structure as a landmark.

The student *t*-test was selected as the appropriate statistical test for analyzing the data in this study. It was assumed that the distribution of the data followed a normal distribution. To determine statistical significance, a significance level of *p* < 0.05 was chosen.

## 3. Results

**Figure 3:**
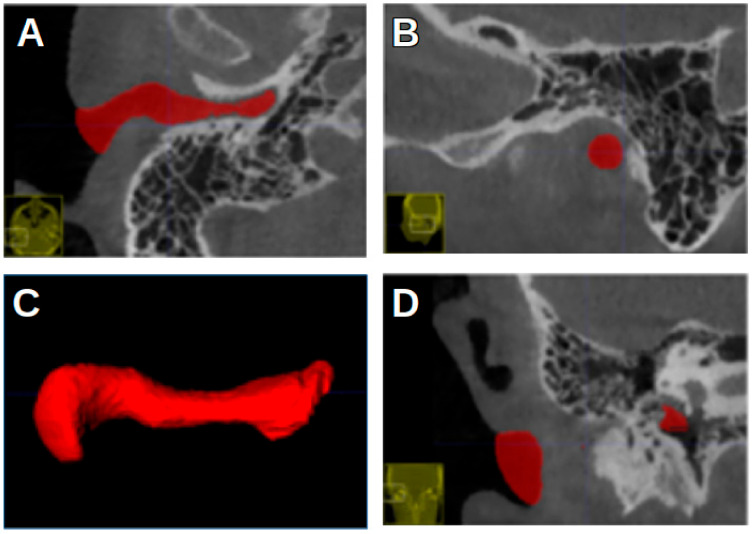
ITK-SNAP window. (**A**) Axial view. (**B**) Sagittal view. (**C**) Three-dimensional rendering of acoustic meatus. (**D**) Coronal view.

Figure 3 shows the ITK-SNAP window with segmentation, with the axial view shown in the upper-left image. In the first four landmarks, the crosshairs were placed such that they fell inside the segmentation of the acoustic meatus.

Per subject, the segmented rendering of the EAM had five features that could be treated as landmarks: the most anterior point (MAP), the most posterior point (MPP), the most superior point (MSP), the most inferior point (MIP), and the epitympanic notch (EN) (Table 1), a structure at the end of the EAM where the tympanic membrane attaches to the bone on the upper side. These features were selected to assess the accuracy with which the examiner had identified reliable and reproducible points that would serve as a replacement for the Porion.

**Figure 4:**
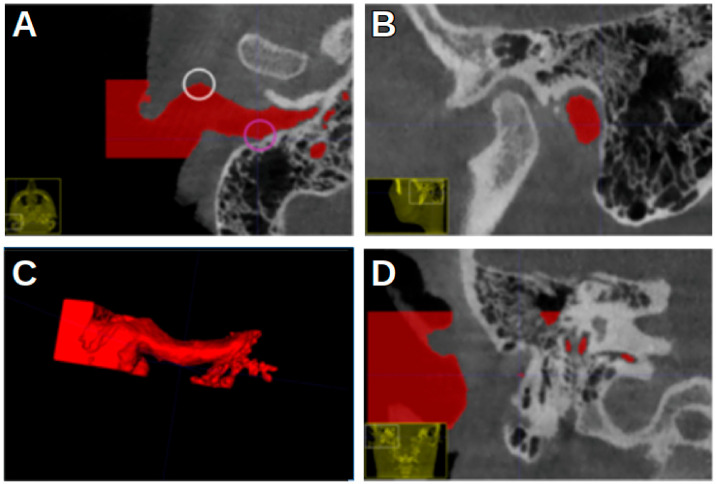
An ITK-SNAP window with a CBCT scan of a left acoustic meatus with red segmentation. (**A**) Axial view. (**B**) Sagittal view. (**C**) Three-dimensional segmentation. (**D**) Coronal view. White circle: the most anterior point of the segmentation. Pink circle: the most posterior point.

**Figure 5:**
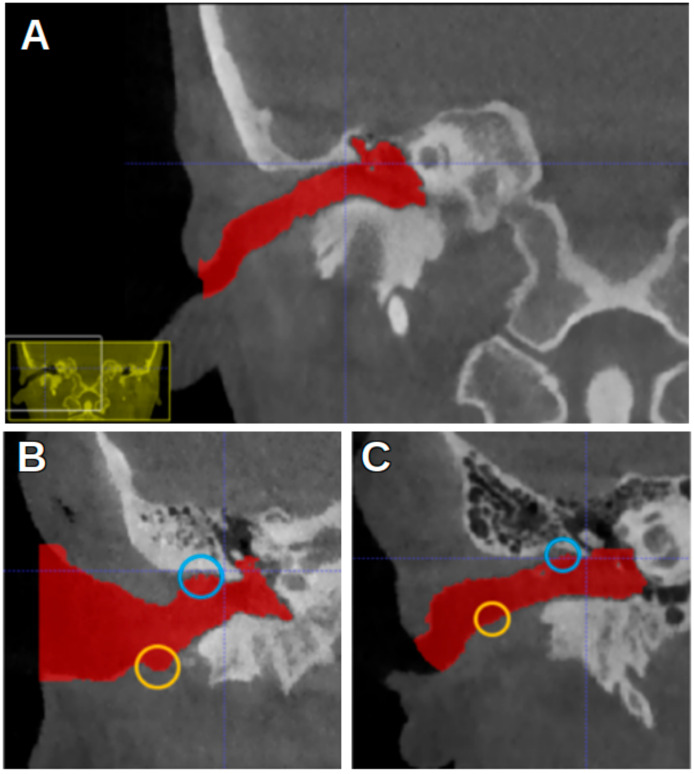
Differences in curvature of EAM. (**A**) CBCT image overview, from coronal view. (**B**) Curved EAM. (**C**) Straight EAM. Blue circles: most superior point of segmentation. Yellow circles: most inferior point.

Figure 4 shows the most anterior and the most posterior point, marked with a white circle and a pink circle, respectively. Figure 5 shows the most superior and the most inferior point, marked with a blue circle and a yellow circle, respectively. In Figure 2, the EN is indicated by a red arrow.

The absolute differences between measurements one and two on the lefthand and righthand sides were calculated for 26 subjects, five landmarks, and three axes. In total, this means that there were 1560 measurements and 780 absolute differences.

**Figure 6:**
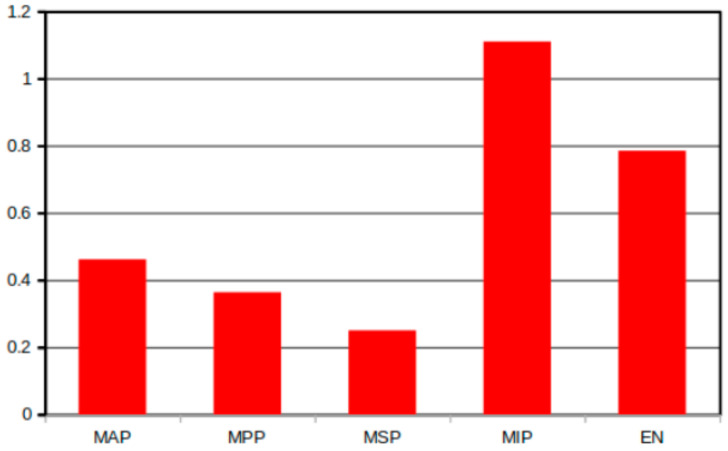
Absolute mean difference per landmark. Scale in millimeters. MAP: most anterior point, MPP: most posterior point, MSP: most superior point, MIP: most inferior point, and EN: epitympanic notch.

Per landmark, Figure 6 shows the mean difference between the first and second measurements. The standard deviation of this difference was also calculated. For the most anterior point, the absolute mean difference between the first and second measurements was 0.46 mm (SD 0.75); for the most posterior point, it was 0.36 mm (SD 0.44). For the most superior point, the absolute mean difference was 0.25 mm (SD 0.26), and for the most inferior point, it was 1.11 mm (SD 1.03). For the epitympanic notch, it was 0.78 mm (SD 0.57). Almost all of the mean differences were less than 1.0 mm; the only landmark at which the difference lay greater than this value was the most inferior point. The most superior point showed the smallest mean difference and standard deviation between the two measurements.

The results indicate that there were no statistically significant differences in the consistency between the most anterior point (MAP) and the most superior point (MSP) (*p* = 0.1851). Similarly, there were no statistically significant differences between the most posterior point and the most superior point (*p* = 0.2641). This implies that it is not possible in this study to differentiate or perceive the MSP differently from the MIP and EN. This suggests that there might be some distinguishing characteristics or factors associated with the MSP that set it apart from the MIP and EN.

However, a statistically significant difference was observed when comparing the recognition of the most superior point (MSP) with two other points, namely the MIP and EN. This difference between the MSP and MIP had a *p*-value of 0.0002, and the comparison between the MSP and EN had a *p*-value of 0.0001.

**Figure 7:**
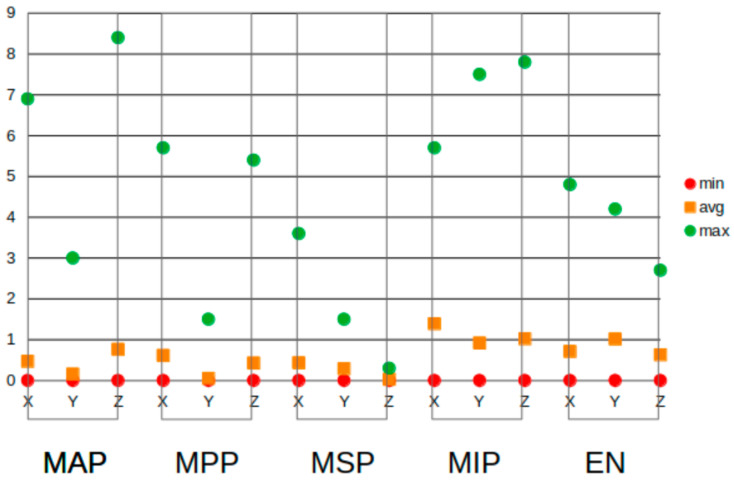
Minimum (red dots), maximum (green dots), and mean absolute (orange squares) differences per landmark and per axis. MAP: most anterior point, MPP: most posterior point, MSP: most superior point, MIP: most inferior point, and EN: epitympanic notch.

Figure 7 shows the absolute mean differences per axis and per landmark. For nearly all landmarks, these differences were less than 1.0 mm. For the most inferior point, the mean differences were 1.39 mm on the X-axis and 1.02 mm on the Z-axis. The mean difference on the Y-axis for the epitympanic notch was 1.02 mm. This figure also shows that in almost all of the landmarks, there were outliers in the measurements. Nearly all landmarks had an outlier that was above 3 mm, the greatest being 8.4 mm on the Z-axis of the most anterior point. The outliers explain the higher standard deviations, which were exceptionally large for the most anterior and most inferior points, where the maximum differences were also the largest. At 0.3 mm, the most superior point on the Z-axis had the lowest maximum, meaning that the maximum difference between the first and second measurements was only one voxel size for this landmark and axis. The minimum difference for all of the landmarks was 0 mm.

**Figure 8:**
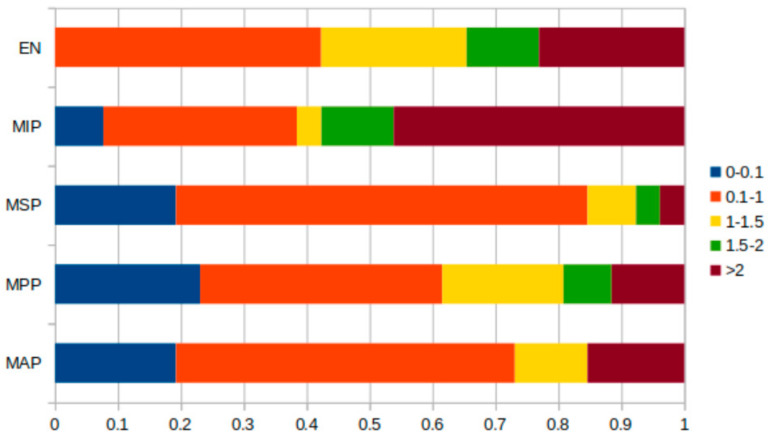
Absolute differences per measurement divided into five groups: MAP: most anterior point, MPP: most posterior point, MSP: most superior point, MIP: most inferior point, and EN: epitympanic notch.

Figure 8 shows the distribution of the measured differences. The differences between the first and second measurements are divided into five groups: those that were between 0 and 0.1 mm; those that were more than 0.1 mm but less than 1.0 mm; those that were more than 1.0 mm but less than or equal to 1.5 mm; those that were more than 1.5 mm but less than 2.0 mm; and those that were more than 2.0 mm. For the most anterior landmark, the graph shows that 19.2% of the measurements were between 0 and 0.1 mm, and 53.8% of the differences were greater than 0.1 but less than 1.0 mm. Only 11.5% of the differences were larger than 1.0 mm, with 15.3% of them being higher than 2.0 mm. The most posterior point had the most differences and 23.0% of the measurements were between 0 and 0.1 mm. An amount of 38.4% of measurements were greater than 0.1 mm but less than 1.0 mm, 19.2% were between 1.5 and 2 mm, 7.6% were between 1.5 and 2 mm, and 11.5% were greater than 2.0 mm. At the most superior point, 19.2.% of the measured differences lay between 0 and 1.0 mm, and 65.3% were less than 1 mm. Only 7.0% of the differences were between 1 and 1.5 mm, and 3.0% were between 1.5 and 2 mm and greater than 2 mm. At 46.1%, the highest number of differences greater than 2.0 mm concerned the most inferior point; 7.0.% were between 0 and 0.1 mm, and 30.7% were greater than 0.1 mm but lower than 1.0 mm. The epitympanic notch was the landmark with the fewest measurement s between 0 and 0.1 mm. This was the landmark, together with the most anterior point and the most superior point, for which most measurements, 42.3%, were in the second group (0.1 < 1.0 mm); and together with the most inferior point, 23.0% of measurements were in the last group (>2 mm).

We used a student t-test with a significance level of *p* < 0.05 to establish the reliability of the measurements in three-dimensional images of five landmarks in the right and left external acoustic meatus.

The present findings highlight that the reliability of the most anterior point (MAP) and the most superior point (MSP) showed no statistically significant differences. The same was observed for the comparison between the most posterior point (MPP) and the most superior point (MSP). In contrast, statistically significant differences were observed with respect to the recognition of the most superior point (MSP) compared to the MIP and EN. This indicates that the study found no significant distinction between the MSP and MAP. Similarly, no significant differences were observed between the MSP and the MPP. In other words, participants’ recognition abilities did not show any significant variation when it came to distinguishing the MSP from the MAP or the MPP.

## 4. Discussion

In this study, the mean differences between the two measurements of the same landmark were found to be less than 1.11 mm. The landmarks were the most anterior point (MAP), the most posterior point (MPP), the most superior point (MSP), the most inferior point (MIP), and the epitympanic notch (EN). Although a landmark in 2D cephalometry is considered precise when the measurement error is less than 1.0 mm, it is debatable whether this standard applies to 3D cephalometry, which has three axes rather than two. Research on 3D cephalometry considered average differences ranging from 2 mm to 1.64 mm. Other studies even found that most differences between landmark measurements were less than 1.5 mm and often less than 1.0 mm.

If a limit of 1.0 mm is considered precise, the most inferior point would be the only landmark that does not meet this criterion. This is in agreement with the study by Schlicher et al. [12], where the mean difference between measurements was used as a measure of consistency and the standard deviation as a measure of precision. They found that the best landmark in terms of both consistency and accuracy was the most superior point, followed by the most posterior point. While the third most consistent landmark was the most anterior point, the third most precise was the epitympanic notch. This landmark is associated with the notch-like border between the EAM and the epitympanic recess, which contains the most superior portions of the malleus and the incus (hearing ossicles). The most inferior point was both the least precise and the least consistent landmark.

A statistically significant difference was observed between the most superior point and both the most inferior point and the epitympanic notch (*p* < 0.05). This finding provides compelling evidence supporting the reliability of the most superior point as a prominent landmark in three-dimensional images. The significant difference suggests that the most superior point possesses distinct characteristics or positional attributes that set it apart from the most inferior point and the epitympanic notch.

**Figure 9:**
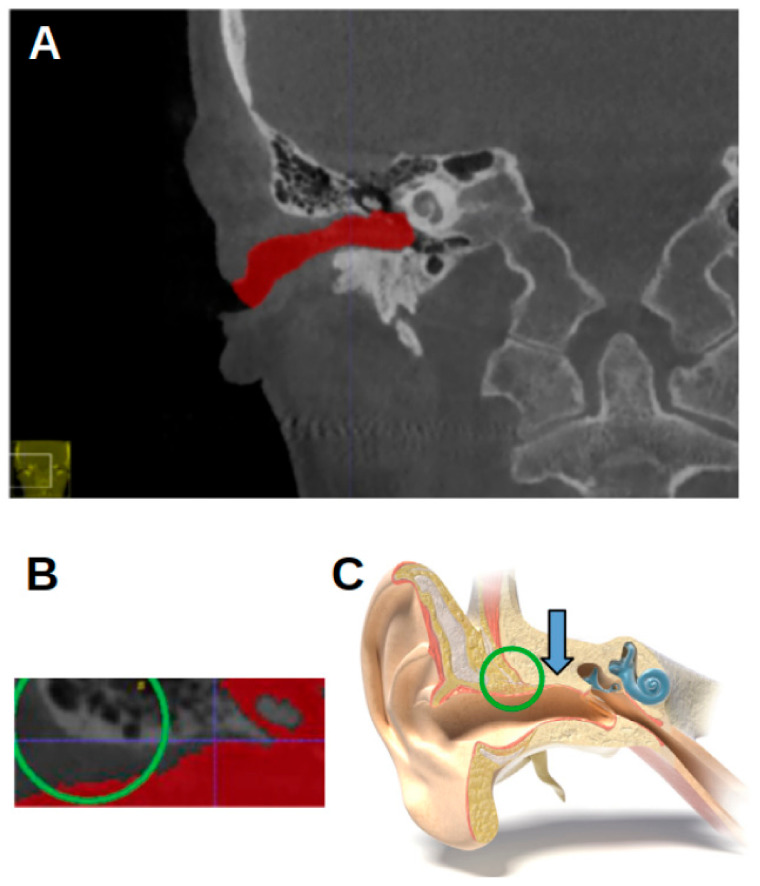
Location of most superior point. (**A**) CBCT image in coronal view. Blue crosshairs: most superior point. (**B**) Green circle: location of most superior point of EAM according to some studies. (**C**) Schematic image (“MedicalGraphics–Drawing Ear anatomy–no labels” at AnatomyTOOL.org by www.MedicalGraphics.de; license: Creative Commons Attribution-NoDerivatives). Green circle: location of most superior point of EAM according to some studies. Blue arrow: location of most superior point according to present study.

Although the most superior point is often used as a definition for the Porion or as a landmark in the EAM, its location was set differently in this study (Figure 9), using the most superior point in the coordinate system of the segmentation.

The location of the landmarks of the most inferior point, due to the curvature, and the epitympanic notch, due to the small area to be segmented, presented some difficulties, making them challenging to pinpoint accurately. The CBCT images used were chosen randomly from a jaw hypermobility study and can be considered representative of a normal population. Inside the Petrous part of the Temporal bone, however, it was not clear which was the most inferior point. As the canals simply descended from the beginning of the EAM to the concha, it was difficult to decide which point was the lowest. This explains why Figure 7 shows a relatively large number of measurements greater than two millimeters.

The epitympanic notch was chosen because of its pointy shape, which could have made it easier to reproduce. Due to this shape, the segmentation sometimes had trouble forming around it, causing location errors. Unlike the other landmarks, which were relatively easy to find because of the segmentation, it was relatively difficult to find the EN, especially in the vertical direction.

**Figure 10:**
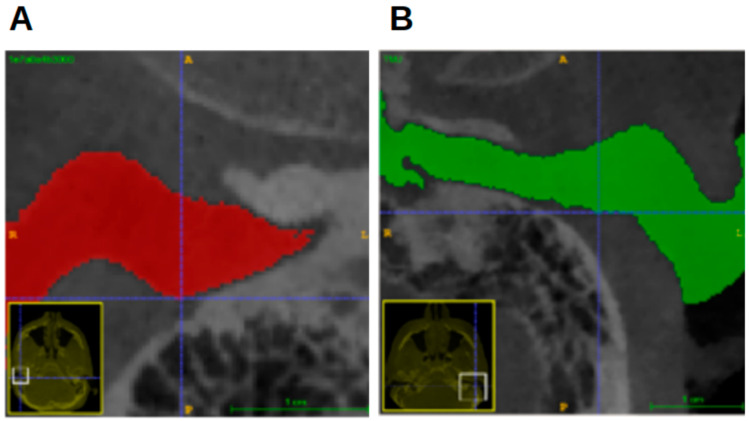
The differences in the location of the most posterior points in an axial view. The blue crosshairs mark the location that is assumed to be the most posterior point. (**A**) The left EAM. (**B**) The right EAM.

The most inferior point was not clear in all individuals (Figure 10). Although the lowest is clear if the EAM is curved, this curve is not always prominent, which makes it more challenging to locate the lowest point, especially inside the Petrous part of the Temporal bone.

The present study was based on automated segmentation of CBCT scans. Segmentation isolates anatomical structures, making them more distinct and easier to visualize. The advantage of this method is that extreme and curved anatomical features can be recognized unambiguously. However, CBCT does not always discriminate perfectly between bone and air [17]. This is especially relevant to the MAP, which may be outside the bone structure. When a surrounding bone structure is present, ITK-SNAP differentiates better between different tissues, as the ear meatus contrasts more clearly with bone than with other tissues. For this reason, it might have been better if, rather than applying a standardized upper threshold (3.0 HU) [18], the threshold had been optimized per scan. It is also the case in CBCT imaging that the position of a voxel in the scan affects its grayscale value. This means that structures with the same density can have a different gray value if they are located in different positions.

Cephalometric landmarks are often considered to be on the border of bony structures, but their exact locations can vary. They are sometimes identified within broader anatomical regions rather than at specific borders, making it sometimes questionable whether a landmark could be located adjacent to bone. Since the slice that should have contained the landmark lacked the surrounding bone, it was difficult to decide the correct position of a landmark, thereby increasing the chance of mistakes. This was the case for the MAP relatively often, and once for the MPP. It could also have been the cause of a number of outliers. However, if segmentations were made twice instead of once, further research could achieve more precise reproducibility. To identify differences, CBCT images are usually made twice and then compared. If the segmentations of the two images are not consistent, and if the differences are too great, the landmarks will not be compatible because they are based on the segmentation.

## 5. Conclusions

A reproducible anatomical landmark in the acoustic meatus in CBCT imaging was identified as an alternative to the Porion in 3D cephalometry. Except for the most inferior point, almost all of the chosen landmarks were reproducible. The most superior point was the most consistent and accurate landmark. To establish whether this method of locating landmarks can be used in practice, further research on the consistency of the segmentation process is required. Utilizing this new reference point enables clinicians to reduce the field of view of the image scans, thereby decreasing patient exposure to radiation and minimizing the uncertainties associated with locating the Porion point.

## Figures and Tables

**Table 1:** Description of five chosen landmarks and how they are located in ITK-SNAP.

	Positioning Method
Most anterior point (MAP) marked with a white circle	The viewpoint of the landmark is adjusted first from the axial view, then from the sagittal view, and finally from the coronal view.
Most posterior point (MPP) marked with a pink circle	Same as for the MAP.
Most superior point (MSP) marked with blue circles	The viewpoint of the landmark is adjusted first from the coronal view, then from the sagittal view, and finally from the axial view.
Most inferior point (MIP) marked with yellow circles	Same as for the MSP and EN.
Epitympanic notch (EN) marked with a red arrow (Figure 2)	Same as for the MSP and MIP.

## Data Availability

The data supporting this study’s findings are available from the corresponding author on reasonable request.

## References

[1] Scarfe W.C., Li Z., Aboelmaaty W., Scott S.A., Farman A.G. (2012). Maxillofacial cone beam computed tomography: Essence, elements and steps to interpretation. Aust. Dent. J..

[2] Mozzo P., Procacci C., Tacconi A., Tinazzi Martini P., Bergamo Andreis I.A. (1998). A new volumetric CT machine for dental imaging based on the cone-beam technique: Preliminary results. Eur. Radiol..

[3] Arai Y., Tammisalo E., Iwai K., Hashimoto K., Shinoda K. (1999). Development of a compact computed tomographic apparatus for dental use. Dentomaxillofac. Radiol..

[4] Proffit W., Fields H., Sarver D., Ackerman JProffit W., Fields H., Sarver D., Ackerman J. (2013). Orthodontic diagnosis: The problem-oriented approach. Contemporary Orthodontics.

[5] Sanders-Mello F., de Menezes L.M., Puetter U.T., Azeredo F., Griekspoor T.C.A., de Windt S., Livas C., Jonkman R.E.G., Rozema F.R., Koolstra J.H. (2023). Acta Plane—A New Reference for Virtual Orientation of Cone Beam Computed Tomography Scans: A Pilot Study. Appl. Sci..

[6] Pittayapat P., Jacobs R., Bornstein M.M., Odri G.A., Lambrichts I., Willems G., Politis G., Olszewski R. (2018). Three-dimensional Frankfort horizontal plane for 3D cephalometry: A comparative assessment of conventional versus novel landmarks and horizontal planes. Eur. J. Orthod..

[7] Paulsen F.W.J., Paulsen F., Waschke J. (2011). Sobotta. Atlas van de Menselijke Anatomie. Deel 3. Hoofd, Hals en Neuroanantomie. (4e druk) Houten, Nederland: Bohn Stafleu van Loghum.

[8] Lagravère M.O., Low C., Flores-Mir C., Chung R., Carey J.P., Heo G., Major P.W. (2010). Intraexaminer and interexaminer reliabilities of landmark identification on digitized lateral cephalograms and formatted 3-dimensional cone-beam computerized tomography images. Am. J. Orthod. Dentofac. Orthop..

[9] Hassan B., Nijkamp P., Verheij H., Tairie J., Vink C., van der Stelt P., van Beek H. (2013). Precision of identifying cephalometric landmarks with cone beam computed tomography in vivo. Eur. J. Orthodontics..

[10] Ludlow J.B., Gubler M., Cevidanes L., Mol A. (2009). Precision of cephalometric landmark identification: Cone-beam computed tomography vs conventional cephalometric views. Am. J. Orthod. Dentofac. Orthopedics..

[11] Lagravè Re M.O., Gordon J.M., Guedes I.H., Flores-Mir C., Carey J.P., Heo G., Major P.W. (2009). Reliability of Traditional Cephalometric Landmarks as Seen in Three-Dimensional Analysis in Maxillary Expansion Treatments. Angle Orthod..

[12] Schlicher W., Nielsen I., Huang J.C., Maki K., Hatcher D.C., Miller A.J. (2012). Consistency and precision of landmark identification in three-dimensional cone beam computed tomography scans. Eur. J. Orthod..

[13] Sanders-Mello F., Jonkman R.E.G., Atay J., Atay J., Rozema F.R., Koolstra J.H. (2024). Symmetry of the external acoustic meatus: A potential alternative reference plane for three-dimensional imaging in dentistry. Heliyon.

[14] Tuijt M., Parsa A., Koutris M., Berkhout E., Koolstra J.H., Lobbezoo F. (2018). Human jaw joint hypermobility: Diagnosis and biomechanical modelling. J. Oral. Rehabil..

[15] Yushkevich P.A., Piven J., Hazlett H.C., Smith R.G., Ho S., Gee J.C., Gerig G. (2006). User-guided 3D active contour segmentation of anatomical structures: Significantly improved efficiency and reliability. Neuroimage.

[16] Lisboa C.d.O., Masterson D., Motta A.F.J., Motta A.T. (2015). Reliability and reproducibility of three-dimensional cephalometric landmarks using CBCT: A systematic review. J. Appl. Oral. Sci..

[17] Fuyamada M., Shibata M., Nawa H., Yoshida K., Kise Y., Katsumata A., Ariji E., Goto S. (2014). Reproducibility of maxillofacial landmark identification on three-dimensional cone-beam computed tomography images of patients with mandibular prognathism Comparative study of a tentative method and traditional cephalometric analysis. Angle Orthod..

[18] Friedli L., Kloukos D., Kanavakis G., Halazonetis D., Gkantidis N. (2020). The effect of threshold level on bone segmentation of cranial base structures from CT and CBCT images. Sci. Rep..

